# Supplementary material for: Hydroxyl Radical vs. One-Electron Oxidation Reactivities in an Alternating GC Double-Stranded Oligonucleotide: A New Type Electron Hole Stabilization
Source: Biomolecules. 2023 Oct 8;13(10):1493. doi: 10.3390/biom13101493 (PMC10605094; doi:10.3390/biom13101493)
Supplement: Supplementary file 1 [file biomolecules-13-01493-s001.zip › biomolecules-2619909-supplementary.pdf]

– SUPPLEMENTARY MATERIALS –

Hydroxyl Radical vs. One-Electron Oxidation Reactivities in an Alternating GC Double-Stranded Oligonucleotide: A New Type Electron Hole Stabilization

Annalisa Masi,[†,$] **Amedeo Capobianco**,[§] **Krzysztof Bobrowski**,[#] **Andrea Peluso**,[§] **and Chryssostomos Chatgilialoglu**[*,†,¶]

[†] Istituto per la Sintesi Organica e la Fotoreattività, Consiglio Nazionale delle Ricerche, 40129 Bologna, Italy;
[$] Istituto di Cristallografia, Consiglio Nazionale delle Ricerche, Via Salaria km 29.300, 00015 Monterotondo, Roma, Italy;
[§] Dipartimento di Chimica e Biologia "A. Zambelli", Università di Salerno, 84084 Fisciano, Italy;
[#] Centre of Radiation Research and Technology, Institute of Nuclear Chemistry and Technology, 03-195 Warsaw, Poland;
[¶] Center for Advanced Technologies, Adam Mickiewicz University, 61-614 Poznań, Poland
* Corresponding Author: Email: chrys@isof.cnr.it.

Synthesis and Purification of 5′-d(GCGCGC)-3′

The ODN single-strand 5′-d(GCGCGC)-3′ was prepared by automated synthesis using the DMT- and the β-(cyanoethyl) phosphoramidite method, on CPG supports (500 Å), with an Expedite 8900 DNA synthesizer (Applied Biosystems, Monza MB, Italy) at 1 μmol scale. The synthesized ODN was cleaved from the solid support and deprotected by the method of two syringes, using AMA reagent [NH_4OH (30%)/CH_3NH_2 (40%) 1:1] for 10 min at room temperature followed by 15 min at 55 °C. The solvent was then removed in a Speedvac (Thermo Fisher Scientific, Monza MB, Italy). The crude 5′-DMT-on oligomer was purified and detritylated on-column by RP-HPLC (Grace Vydac C18 column 5 μm, 50 x 22 mm, Thermo Fisher Scientific, Rodano (MI), Italy). The ODN was further purified by SAX HPLC (preparative DNA Pac PA-100 column, 13 μm, 22 x 250 mm, Thermo Fisher Scientific, Rodano (MI), Italy) with TRIS HCl 25 mM, pH = 8.0 (buffer A) and TRIS HCl 25 mM, $NaClO_4$ 0.5 M, pH = 8.0 (buffer B) at a flow rate of 9 mL/mineluting with 2-30 % B in 30 min, 30% B for 10 min, then 30-40% B in 5 min monitoring at 254 nm. The purified ODN fractions were concentrated, desalted on Waters SepPakTM-C18-cartridges (Sesto San Giovanni (MI), and lyophilized. The final yield was estimated by UV absorption in aqueous solution at 260 nm by a Cary 100 UV/Vis Spectrometer (Agilent, Cernuscosul Naviglio (MI), Italy). The purity of the ODN was determined by analytical SAX HPLC chromatography (DNA Pac PA100, 13 μm, 4 x 250 mm, Thermo Fisher Scientific, Rodano (MI), Italy) (Figure S1).

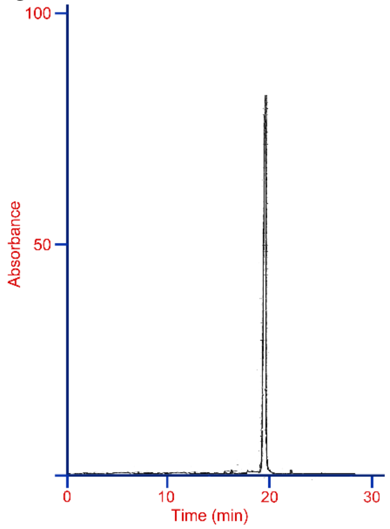

Figure S1. Analitycal SAX HPLC chromatograms of purified ODNs. Column and conditions: SAX DNAPac PA-100 column, 5μm, 4x250 mm; mobile phase A: TRIS.HCl 25 mM, pH 8.0, mobile phase B: TRIS.HCl 25mM, $NaClO_4$ 0.5M, pH 8.0. Gradient: 2-30 % B in A in 30 min. Flow rate was 1 mL/min.

Preparation of ds-ODN and Melting Point Experiments

To obtain the ds-ODN, the single strand 5′-d(GCGCGC)-3′ was annealed in buffer solution containing 50 mM sodium phosphate (NaH_2PO_4). A set of UV melting measurements were carried out to investigate the relative stability of ds-ODN under buffer solutions containing different sodium ion concentrations and minimizing the presence of chloride ions (Cl^-). It is well-known that Cl^- reacts quite fast with $HO^•$ in aqueous solution producing $Cl^•$ and HO^-. To overcome this problem and in order to find the best condition to ensure the stability of ds-ODN, reducing the concentration of chloride ions, different buffers were tested (see Table S1). The substrates were constructed by heating the two strands of the substrates at 90 °C for 10 min and

subsequently allowing the temperature to drop slowly to room temperature (25 °C). The melting curves were determined by monitoring the absorbance of the solutions at 260 nm as a function of temperature. A Cary 100 spectrophotometer system equipped with a 1 mL quartz cuvette with a 1 cm path length was used for the measurements. The temperature cycles from 20 to 80 °C per experiment were recorded with a temperature controller using a 0.3 °C/min heating rate. The recorded T_m values are reported in Table S1, which represent the temperature at which 50% of the ds-ODN is unpaired. The higher its value the more stable the duplex.

Table S1. Melting points (T_m) of the hexamer ds-ODN in different buffer conditions

Entry	5′-d(GCGCGC)-3′ duplex	T_m (°C)
1	NaH_2PO_4 (100 mM)	45.9
2	NaH_2PO_4 (50 mM)	45.9
3	NaH_2PO_4 (10 mM)	36.3
4	NaH_2PO_4 (100 mM) + NaCl (100 mM)	46.5
5	NaH_2PO_4 (100 mM) + NaCl (20 mM)	41.5

In Figure S2 the comparison of different melting curves of ds-ODN is reported. The comparison for 100 mM vs. 50 mM of NaH_2PO_4 buffers (entries 1 vs 2) indicates the same T_m and very similar melting curves (Figure S2A). Analyzing the area around 20 °C, a different absorbance is observed, where the ds-ODN percentage of 82% was calculated for entry 1 versus 87% for entry 2, highlighting a greater stability for the latter condition. In Figure S2B, the 50 mM NaH_2PO_4 buffer is compared to 100 mM NaH_2PO_4 + 100 mM NaCl, where the T_m of the latter is slightly higher (ΔT = 0.6 °C). Analyzing the areas around 20 °C, similar percentages of duplex are calculated (87% for entry 2 vs 91% for entry 4), while between 20-30 °C the absorbance of the blue curve increases, due to the hyperchromic effect, whereas the absorbance of the pink curve does not change, indicating a greater stability.

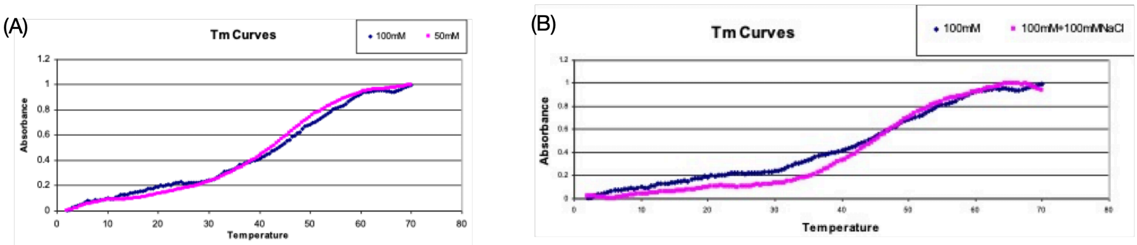

Figure S2. Melting curves of ds-ODN: (**A**) 100 mM NaH_2PO_4 (blue) vs. 50 mM NaH_2PO_4 (pink); (**B**) 100 mM NaH_2PO_4 (blue) vs. 100 mM NaH_2PO_4 + NaCl (100 mM) (pink).

Theoretical Calculations

Table S2. Predicted energy changes (ΔE, kcal/mol) for addition of $SO_4^{\bullet-}$ to $^{me}G{:}C^{me}$ pair in water; spectroscopic properties, wavelengths and oscillator strength, of the radical adducts in water.

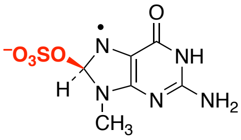 ΔE = -12.8		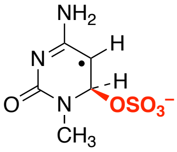 ΔE = -5.6		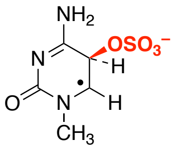 ΔE = -4.6	
λ, nm	f	λ, nm	f	λ, nm	f
434	0.015	430	0.052	799	0.003
397	0.021	347	0.018	619	0.015
390	0.002	308	0.020	480	0.020
377	0.041	293	0.013	400	0.012
355	0.002				
336	0.010				
329	0.001				
324	0.003				
320	0.017				
312	0.088				
307	0.027				
303	0.211				

Table S3. Comparative analysis of predicted (PCM/TD-B3LYP/TZVP) UV/vis transitions of $^{me}G^{\bullet+}$ and $[^{me}G{:}C^{me}]^{\bullet+}$

Transition of $[^{me}G{:}C^{me}]^{\bullet+}$			Corresponding transition of $^{me}G^{\bullet+}$	
λ / nm	osc. stre.	assignment	λ / nm	osc. stre.
918	6.3 x 10^{-3}	[a] CT, π(C) → π*(G)	–	–
664	2.2 x 10^{-3}	CT, π(C) → π*(G)	–	–
529	6.7 x 10^{-3}	π(G) → π*(G)	544	1.0 x 10^{-2}
499	3.2 x 10^{-2}	π(G) → π*(G)	462	2.8 x 10^{-2}
374	0.112	π(G) → π*(G)	372	0.114
295	1.1 x 10^{-3}	CT, π (C) → π*(G)	–	–
293	0.127	π(G) → π*(G) and π(C) → π*(C)	290	0.104
266	0.134	π(G) → π*(G) and π(C) → π*(C)	271	0.189
263	0.174	π(G) → π*(G) and π(C) → π*(C)	–	–

[a]CT is "charge transfer" abbreviated.

Table S4. Comparative analysis of predicted (PCM/TD-B3LYP/TZVP) transitions of $[^{me}G{:}C^{me}]^{\bullet+}$ and $[^{me}G{:}C^{me}/^{me}C{:}G^{me}]^{\bullet+}$

Transition of $[^{me}G{:}C^{me}/^{me}C{:}G^{me}]^{\bullet+}$			Corresponding transition of $[^{me}G{:}C^{me}]^{\bullet+}$	
λ / nm	osc. stre.	assignment	λ / nm	osc. stre.
1839	0.139	[a,b] π(G…G) → π*(G…G)	–	–
1027	8.0×10^{-3}	CT, π(C…C) → π*(G…G)	918	6.3×10^{-3}
955	8.0×10^{-4}	CT, π(C…C) → π*(G…G)	918	6.3×10^{-3}
739	4.1×10^{-3}	CT, π(C…C) → π*(G…G)	918	6.3×10^{-3}
665	1.6×10^{-3}	CT, π(C…C) → π*(G…G)	664	2.2×10^{-3}
565	1.2×10^{-2}	π(G…G) → π*(G…G)	529	6.7×10^{-3}
539	1.7×10^{-3}	π(G…G) → π*(G…G)	529	3.2×10^{-2}
486	6.5×10^{-3}	π(G…G) → π*(G…G)	499	7.7×10^{-3}
[c] 388	9.0×10^{-4}	π(G…G) → π*(G…G)	374	0.112
[c] 381	6.8×10^{-3}	π(G…G) → π*(G…G)	374	0.112
305	9.0×10^{-4}	CT, π(C…C) → π*(G…G)	295	1.1×10^{-3}
302	7.2×10^{-4}	CT, π(C…C) → π*(G…G)	295	1.1×10^{-3}
270	0.064	π(G…G) → π*(G…G)	293	0.127
268	9.0×10^{-3}	π(G…G) → π*(G…G)	266	0.134
260	0.055	CT, π(C…C) → π*(G…G) + π(G…G) → π*(G) + π(G…G) → π*(G…G) + π(C…C) → π*(C…C)	–	–
258	0.014	π(C…C) → π*(C…C) + CT, π(C…C) → π*(G…G) + π(G…G) → π*(G…G) + π(G…G) → π*(G)	–	–
257	0.431	π(C…C) → π*(C…C) + π(G…G) → π*(G) + CT, π(C…C) → π*(G…G)	–	–
[d] 256	0.110	π(G…G) → π*(G…G) + π(G…G) → π*(G) π(C…C) → π*(C…C) + CT, π(C…C) → π*(G…G)	–	–

[a] HOMO-LUMO transition, with both MOs exhibiting electron density equally distributed on both guanines, see Figure S2. [b] G…G and C…C denote the coupled π systems of alternate guanines and cytosines, respectively. [c] Notice the large reduction of intensity with respect to the corresponding transition (λ = 374 nm, f = 0.112) of $[^{me}G{:}C^{me}]^{\bullet+}$. [d] Transitions with oscillator strength less than 1.0×10^{-4} are not reported.

Table S5. Predicted (PCM/TD-CAM-B3LYP/TZVP) low energy transitions of $[^{me}G{:}C^{me}/^{me}C{:}G^{me}]^{\bullet+}$

λ / nm	osc. stre.	assignment
6263	0.17	[a] π(G…G) → π*(G…G)
612	5.8×10^{-3}	CT, π(C…C) → π*(G…G)
591	1.3×10^{-3}	CT, π(C…C) → π*(G…G)
549	1.2×10^{-2}	CT, π(C…C) → π*(G…G)
522	7.8×10^{-3}	π(G…G) → π*(G…G)

[a] HOMO-LUMO transition, with both MOs exhibiting electron density equally distributed on both guanines, see Figure S2.

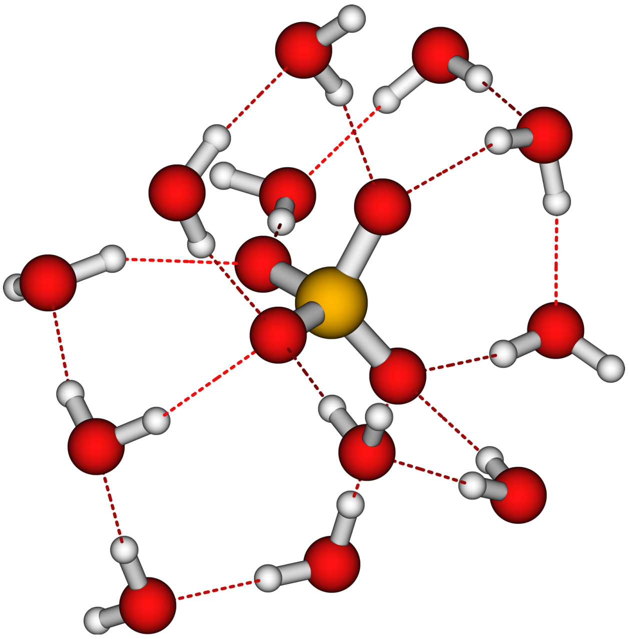

Figure S3. Optimized geometry (PCM/B3LYP/6-311++G**) of $SO_4^{\bullet-}$ radical anion including its first sovation shell.

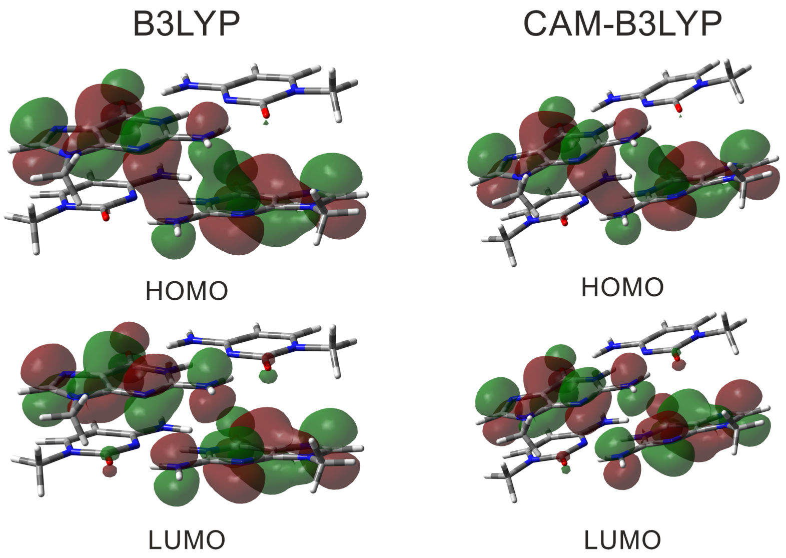

Figure S4. Isosurface contour plots of the HOMO and LUMO of $[^{me}G{:}C^{me}/^{me}C{:}G^{me}]^{\bullet+}$ in both levels of theory (B3LYP and CAM-B3LYP).
